# School- and Family-Level Socioeconomic Status and Health Behaviors: Multilevel Analysis of a National Survey in Wales, United Kingdom

**DOI:** 10.1111/josh.12242

**Published:** 2015-03-02

**Authors:** Graham F Moore, Hannah J Littlecott

**Affiliations:** a DECIPHer, School of Social Sciences, Cardiff University, 1-3 Museum PlaceCardiff CF10 3BD, United Kingdom; b DECIPHer, School of Social Sciences, Cardiff University, 1-3 Museum PlaceCardiff CF10 3BD, United Kingdom

**Keywords:** socioeconomic status, inequalities, adolescent, health behavior

## Abstract

**Background:**

Interventions to address inequalities in adolescent health behaviors often target children from less affluent families, or schools in poorer areas. Few studies have examined whether school- or family-level affluence predicts health behaviors independently, or in combination.

**Methods:**

This article reports secondary analysis of the Welsh Health Behavior in School-aged Children (HBSC) survey. Mixed-effects logistic regression models test associations of school and family socioeconomic status (SES) with smoking, fruit and vegetable consumption, alcohol consumption, and physical activity.

**Results:**

Higher family SES was associated with healthier behaviors, except in relation to alcohol consumption. For all behaviors except physical activity, school-level SES was independently associated with healthier behaviors. In higher SES schools, a stronger association of family SES with health behavior was observed, particularly in relation to smoking and physical activity.

**Conclusions:**

School and family SES may exert independent and combined influences upon adolescent health behaviors. Targeting interventions toward deprived schools may fail to address substantial inequalities within more affluent schools. Targeting deprived families may fail to address behaviors of children from affluent families, attending more deprived schools. Identifying universal health improvement interventions which have greater effects among children from poorer backgrounds may be a more effective means of reducing inequalities.

Lower socioeconomic status (SES) is associated with increased mortality and morbidity throughout the life-course.^1^,^2^ Health inequalities are driven by a range of material factors, as well as psychosocial factors associated with relative social status.^3^ Behavioral factors such as smoking, unhealthy diets, and physical inactivity also contribute to health inequalities, with less healthful behaviors typically observed among lower SES groups.^4^,^5^ Adolescence is a critical life-course period during which patterns of health behavior are formed, before tracking into adulthood.^6^,^7^ Although widening into adulthood, there is substantial evidence that socioeconomic inequalities in health behaviors emerge during adolescence. For example, Hanson and Chen's review of studies in developed countries reported consistent associations of SES with smoking, diet, and physical activity, although inconsistent associations with alcohol consumption.^8^ Reducing inequalities in health behaviors during adolescence may play an important role in interrupting the intergenerational reproduction of health inequalities.

School-based health interventions provide opportunities to reach large numbers of children, and range from health education programs,^9^ to holistic settings-based approaches.^10^ Reviews indicate that interventions based on education alone are typically ineffective,^11^ whereas interventions including components at multiple levels often have greater effects.^12^ To date, although the primary focus of evaluation research has been the overall effectiveness of school-based interventions, with more limited attention to their impacts on inequality, school-based interventions have adopted a variety of approaches to targeting lower SES groups. For example, free school meal (FSM) entitlement in the United Kingdom (UK) is offered to children whose families are in receipt of state benefits. By contrast, provision of school breakfasts has either targeted whole schools in more deprived areas^13^ or has been offered on a universal basis.^14^

These approaches perhaps reflect differing assumptions regarding the nature of inequalities. Targeting children from poorer families assumes that family-level socioeconomic factors are key determinants of health behavior. In studies investigating adolescent health behavior, SES almost exclusively has been considered in terms of parental occupation, education, or affluence.^8^ Children from less affluent families may be exposed to more adults who model unhealthy behaviors,^15^ whereas less affluent parents may face structural barriers to providing healthier foods, or opportunities for enjoyable physical activities in which their children will be intrinsically motivated to engage.^16^ Targeting schools with less affluent overall intakes may, in part, reflect assumptions that this is the most efficient means of reaching large proportions of children from poorer families. However, it may also reflect assumptions regarding how the dynamics of school systems are impacted by SES composition.

Much sociological theory points to a need to consider SES as a multilevel construct,^17^ with inequalities shaped by factors at individual levels, within schools and neighborhoods, and larger geographical regions.^18^,^19^ The Theory of Fundamental Causes argues that health inequalities arise from the unequal distribution of flexible resources such as knowledge, money, power, social influence, and status.^20^ As mechanisms linking SES to health are weakened, they are replaced, with individuals or groups with more flexible resources using them to gain access to emerging mechanisms to improve health. Historical mechanisms such as sanitation and infectious disease have been replaced largely by factors such as smoking and obesity.^20^ Both were once more prevalent among higher SES groups, although became stigmatized markers of low SES, as scientific knowledge of their health consequences emerged. Hence, known risk factors for disease often cluster by SES, even when explained by different mechanisms. Parents with more flexible resources may use them to gain access to services that affect children's health, such as schools, or to influence health-related activity within those schools. One study for example indicates that more affluent parents more strongly advocate policies around limiting unhealthy foods in school lunches.^21^ One might expect poorer children within more affluent schools to benefit from changes arising from pressures from more well-resourced parents. However, schools reproduce culture, including the inequalities within it, reflecting values which are a natural extension of affluent home environments.^22^ For children from poorer backgrounds attending schools with more affluent intakes, discrepancy between cultures within and outside of school systems may be amplified, potentially leading to disengagement and rejection of school norms.

The majority of studies examining associations of SES with adolescent health behavior have examined only family-level SES. Some have linked health behaviors to the SES composition of the school the child attends,^14^,^23^,^24^ or the neighborhood in which the child resides.^25^ Few have considered whether these trends reflect compositional effects, or whether school-level SES exerts influences upon behavior which are independent of family SES. Simetin et al^26^ reported independent associations of school-level SES with smoking in a Croatian sample, although by contrast to the dominant trend in most Western countries, smoking rates were higher in children from more affluent families. Mathur et al^25^ studied SES at multiple levels and found lower overall smoking levels, but significantly stronger social gradients within affluent neighborhoods. To our knowledge, no studies have examined how school- and family-level SES interact to predict adolescent health behaviors.

This article uses data from the Health Behavior in School-aged Children (HBSC) in Wales to examine links between SES and the 4 health behaviors included within Hanson and Chen's aforementioned review (smoking, physical activity, fruit and vegetable consumption, and alcohol consumption). It examines whether (1) family affluence or school-level affluence independently predicts health behaviors among 11- to 16-year-old adolescents; and (2) family- and school-level affluence interact in predicting adolescent health behavior.

## Methods

### Participants

The HBSC survey collects self-reported data on health behaviors and well-being from children aged 11-16, through classroom-based questionnaires. Surveys are currently carried out every 4 years by an international network of research teams in collaboration with the World Health Organization Regional Office for Europe.^27^ This article analyzes data from the survey in Wales, for which data were collected between September 2009 and January 2010. Schools were selected, with probability of selection proportional to pupil roll size, from a stratified list of all state maintained (N = 223) and independent (N = 62) secondary schools in Wales. In total, 134 schools were approached, of whom 82 participated. Within each school, 1 class per year group was selected to participate. A total of 9194 children (of 10,077 eligible children) completed the survey.

### Instrumentation

#### Health behaviors

Analyses focus on 4 health behaviors: *smoking*, *alcohol consumption*, *fruit and vegetable consumption*, and *physical activity*. Smoking is measured by asking children: “How often do you smoke tobacco at present?” with response options of “every day,” “at least once a week,” “less than once a week,” or “I don't smoke.” Children were classified as smokers if they gave any response other than “I don't smoke.” Children were asked on how many days in the previous week they participated in at least 60 minutes of physical activity. Children who selected more than 5 days were classed as physically active. According to the UK National Health Service, a balanced diet comprises 5 core components; larger amounts of fruit and vegetables and starchy foods, moderate amounts of dairy products and protein sources, and small amounts of foods high in fats and sugars.^28^ This article focuses on one of these components: fruit and vegetable consumption. Children were asked how often they ate fruits and how often they ate vegetables (never, less than once a week, once a week, 2-4 days a week, 5-6 days a week, once daily, more than once daily). A score of 1 was given if children reported eating fruits or vegetables at least daily, 0 if they did not. Children were also asked how many times they had drunk alcohol in the past 30 days (never, 1-2 times, 3-5 times, 6-9 times, 10-19 times, 20-39 times, or more than 40 times); those who reported drinking 3 or more times in the last month were considered to be regular drinkers. A multiple health behavior index was created by assigning a score of 0 or 1 for each behavior, and summing to create a score from 0 (smoker, regular drinker, nonconsumer of fruit and vegetables, and insufficiently active) to 4 (nonsmoker, nonregular drinker, consumer of fruit and vegetables, and sufficiently active).

#### Socioeconomic status

Measures of SES were (1) *school-level* FSM entitlement (divided into low [<10% of children entitled to FSM], medium [11-19%] and high tertiles >19%); and (2) *family* affluence, using the Family Affluence Scale (FAS).^29^ Free school meals are offered in Wales to children whose parents are in receipt of a range of state benefits such as Income Support. Family Affluence Scale includes 4 items asking children whether they have their own bedroom, how many computers and cars their family own, and how many holidays their family took in the past year. Items are summed to give a total affluence score. Where aggregated at the school level, FAS and FSM entitlements were highly correlated (r = .77).

#### Confounders

Children reported their sex, month and date of birth, and grade (eg, year 7 [11-12 years] to year 11 [15-16 years]). Age was calculated by subtracting date of birth from date of data collection. Schools were classed as village, town, or urban, and due to overrepresentation of children from urban areas in the HBSC survey, urbanization was included as a control variable. Children were also asked to indicate how wealthy the area they lived in was, on a 5-point scale, ranging from “not at all” to “very.”

### Data Analysis

Univariate associations between SES and health behaviors were examined using design adjusted chi-square analyses, comparing proportions of children within high, medium, and low FAS/FSM schools/families classed as smokers, regular drinkers, physically active, and daily consumers of fruits and/or vegetables. Design-based F statistics are reported from these analyses. Intracluster correlations for each health behavior were then estimated using mixed-effects binary logistic regression models, containing only the constant.^30^ For the combined health behavior index, scored 0 to 4, ordinal logistic models were used. The individual-level SES measure was then entered into the model, alongside individual-level confounders (age, sex, urban/rural classification). Intracluster correlations were recalculated. Two sets of models were run with alternative markers of school-level affluence: (1) FSM entitlement (a high score represented low FSM entitlement, or high affluence) and (2) aggregated FAS score. The proportion of school-level variance explained by school-level affluence was calculated for each variable, by examining percentage change in variance between the model containing individual variables only, and models using school-level terms. To evaluate interactions between school- and family-level deprivation, FAS score was set as a random slope, and a cross-level interaction term for FSM × FAS entered. Odds ratios and 95% confidence intervals for school- and family-level SES and their interaction are reported from the final model.

## Results

### Sample Description

Overall, 9194 children completed HBSC. The sample included almost an even split of boys and girls (N = 4594 [50.2% boys]), and an approximately even split of children from each year group (Table1). For all variables except FAS score, less than 5% of data were missing. Family FAS data were available for 8281 (9.9% missing) children. Children who failed to complete FAS did not differ from other children in terms of health behaviors, or school-level FSM entitlement, although younger children and boys were least likely to complete FAS.

**Table 1:** Sample Characteristics for Adolescents Completing the Health Behavior in School-Aged Children Survey in Wales (N = 9194)

	Frequency	Percentage
Sex		
Boy	4594	50.2
Girl	4565	49.8
Grade		
Year 7	1923	20.9
Year 8	2026	22.0
Year 9	1908	20.8
Year 10	1687	18.4
Year 11	1650	18.0
FSM entitlement		
High (19+)	2264	24.6
Medium (10 < 19)	3405	37.0
Low (<10%)	3525	38.3

FSM, free school meal.

### Health Behavior by SES

There was a negative association of school and family SES with smoking, and positive associations of both affluence markers with fruit and vegetable consumption (Table2). For alcohol consumption and physical activity, only family SES was significant. Notably, for alcohol consumption, children from more affluent families report drinking more than those from poorer families.

**Table 2:** Frequency and Percentage of 11- to 16-Year-Old Pupils in Wales Classified as Smokers, Regular Drinkers, Taking Sufficient Physical Activity, and Consuming Fruit and/or Vegetables Daily by Family Affluence Scale (FAS) Score and Free School Meal (FSM) Entitlement

	Smoking	Alcohol	Activity	Fruit and Vegetables
FAS				
Low (N = 834/832/814/843)	90 (10.8)	133 (16.0)	217 (26.7)	285 (33.8)
Medium (N = 3029/3003/2982/3045)	279 (9.2)	615 (20.5)	773 (25.9)	1218 (40.0)
High (N = 4368/4318/4316/4383)	311 (7.1)	902 (20.9)	1312 (30.4)	2147 (49.0)
Design-based F	**8.0**	**5.0**	**10.7**	**39.8**
FSM				
Low (N = 3514/3491/3471/3523)	229 (6.5)	664 (19.0)	988 (28.5)	1871 (53.1)
Medium (N = 3380/3345/3327/3398)	271 (8.0)	663 (19.8)	943 (28.3)	1339 (39.4)
High (N = 2243/2206/2200/2260)	251 (11.2)	470 (21.3)	676 (30.7)	801 (35.4)
Design-based F	**8.0**	0.6	1.3	**32.8**

Associations that are significant (p < .05) are highlighted in bold. Variation in Ns due to small numbers of missing data for specific behaviors.

### School-Level Clustering in Health Behavior

School-level intracluster correlations (ICC) ranged from 0.01 (physical activity) to 0.05 (smoking) in unadjusted models. In models adjusted for individual-level factors, ICCs mostly increased, indicating that compositional differences were causing underestimation of between-school differences. In particular, the ICC for alcohol consumption increased substantially from 0.01 to 0.05. In final models, adjusted for school-level FSM entitlement, most ICCs declined slightly, ranging from 0.01 to 0.06.

### Independent and Combined Associations of School and Family Affluence With Health Behaviors

In the models presented in Table3, family affluence predicts all 4 behaviors. School-level affluence is associated with all behaviors except physical activity in analyses using FSM. For alcohol consumption, associations of school and family affluence operated in opposing directions; children appeared more likely to drink regularly if they were in lower SES schools and came from higher SES families. Where using aggregated FAS score as the measure of school affluence however, associations with alcohol consumption were no longer significant. The proportion of between-school variance explained by school-level affluence was substantially higher for fruit and vegetable consumption (24% using FSM entitlement, 16% using aggregated FAS score) and smoking (13% and 15%) than for physical activity (5% and 4%) and alcohol consumption (2%). For the combined health behavior index, 23% of between-school variance was explained by FSM entitlement, and 19% by aggregated FAS score. There was a consistent interaction between FSM entitlement and family affluence, although only for smoking, physical activity, and the combined health behavior index did this reach significance, with a near significant interaction for fruit and vegetable consumption (p = .07 using FSM; p = .05 using aggregated FAS). Hence, as school affluence increased, overall behaviors became healthier, although within school inequalities widened.

**Table 3:** Odds Ratios and Confidence Intervals From Multilevel Logistic Regression Models Examining Associations of Family Affluence Scale (FAS) Score and School-Level Free School Meal (FSM) Entitlement With Health Behaviors of 11- to 16-Year-Old Children in Wales

		Smoking (N = 7927)	Alcohol (N = 7859)	Activity (N = 7823)	Fruit and Vegetables (N = 7963)	Multiple Health Behavior (N = 7689)
Models using FSM entitlement as school-level measure						
Main effects						
School level	FSM	**0.78 (0.65 to 0.93)**	**0.87 (0.75 to 1.00)**	0.94 (0.85 to 1.03)	**1.39 (1.25 to 1.54) **	**1.27 (1.13 to 1.43)**
Individual-level variables	FAS	**0.88 (0.81 to 0.95)**	**1.08 (1.03 to 1.14)**	**1.13 (1.08 to 1.18)**	**1.15 (1.10 to 1.20)**	**1.08 (1.03 to 1.12)**
	Age	**2.09 (1.94 to 2.25)**	**1.76 (1.68 to 1.85)**	**0.85 (0.82 to 0.88)**	**0.91 (0.88 to 0.94)**	**0.70 (0.67 to 0.72)**
Interaction effects	FSM × FAS	**0.90 (0.83 to 0.98)**	0.95 (0.90 to 1.01)	**1.06 (1.01 to 1.12)**	1.04 (0.99 to 1.10)	**1.08 (1.03 to 1.12)**
ICC—constant only		0.05	0.01	0.01	0.03	0.04
ICC—individual-level variables		0.06	0.05	0.01	0.04	0.04
ICC—level 1 and 2 variables		0.06	0.04	0.01	0.02	0.03
Models using aggregated FAS score as school-level measure						
Main effects						
School level	FAS (mean)	**0.56 (0.38 to 0.82)**	0.81 (0.60 to 1.10)	0.96 (0.79 to 1.17)	**1.78 (1.40 to 2.25)**	**1.57 (1.24 to 1.98)**
Individual-level variables	FAS	**0.92 (0.85 to 0.98)**	**1.10 (1.05 to 1.16)**	**1.09 (1.03 to 1.16)**	**1.14 (1.09 to 1.18)**	**1.09 (1.06 to 1.13)**
	Age	**2.08 (1.94 to 2.24)**	**1.76 (1.68 to 1.85)**	**0.85 (0.82 to 0.88)**	**0.91 (0.88 to 0.94)**	**0.70 (0.67 to 0.72)**
Interaction effects	FAS mean × FAS	**0.75 (0.62 to 0.90)**	0.94 (0.82 to 1.07)	**1.12 (1.00 to 1.25)**	1.11 (1.00 to 1.24)	**1.16 (1.06 to 1.28)**
ICC—2 level model		0.06	0.04	0.01	0.03	0.03

Associations which are significant (p < .05) are highlighted in bold.

ICC, intracluster correlations.

**Figure 1:**
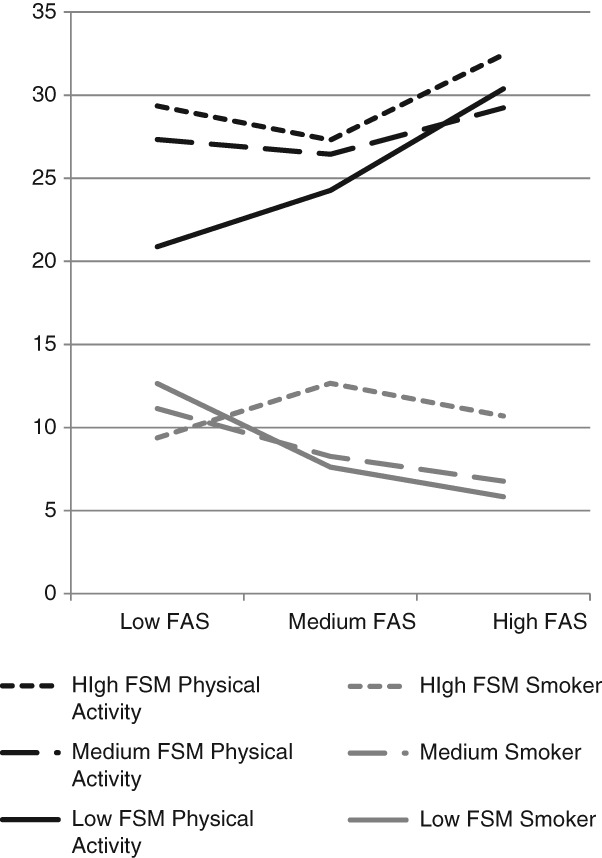
Percentages of Children Classified as Smokers and as Taking Sufficient Physical Activity, by School (Lower FSM = Higher Affluence) and Family (Higher FAS = Higher Affluence) Level Affluence

**Figure 2:**
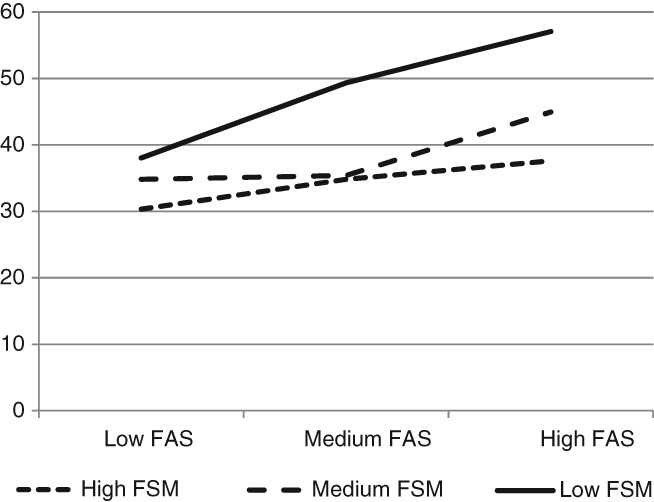
Percentage of Children Eating Fruit and/or Vegetables at Least Daily by School (FSM) and Family (FAS) Level Affluence

The interactions between school- and family-level affluence in terms of smoking and physical activity are portrayed in Figure 1. Each line depicts the gradient in percentages of children smoking or participating in physical activity within schools of (1) high affluence (ie, low FSM entitlement); (2) medium affluence (ie, medium FSM entitlement); and (3) low affluence (ie, high FSM entitlement). For physical activity, children from less affluent families were less physically active if they attended a more affluent (low FSM) school. For children from more affluent families, physical activity levels are similar regardless of school SES. For smoking, there is a steeper socioeconomic gradient in affluent schools. Among children from poorer families, smoking levels are similar regardless of school affluence, whereas for children from more affluent families, smoking is less likely if attending a more affluent school. As data in Table3 indicate, the interaction between school and family SES in relation to fruit and vegetable consumption approached statistical significance. This interaction is depicted in Figure 2, showing that for high, medium, and low FSM schools, fruit and vegetable consumption was highest among children from more affluent families. However, the association between family SES and consumption of fruit and vegetables is stronger in more affluent schools.

## Discussion

Our results indicate that lower family affluence is associated with a higher level of adolescent smoking, physical inactivity, and poorer diet, although lower risk of regular alcohol consumption. These findings are largely consistent with Hanson and Chen's review of studies on SES and adolescent health behavior, which reported consistent associations of SES with physical activity, diet, and smoking, although not alcohol consumption.^8^ Family affluence may contribute to adolescent health behaviors via a range of mechanisms. Health behaviors are commonly learned from parents through modeling processes,^31^ and children from more deprived families are for example more likely to have at least 1 parent who smokes.^15^ Poorer parents may also face barriers to meeting the costs associated with providing physical activity opportunities and healthy foods.^32^ In addition, there is some evidence that children from poorer families are more likely to stay inside due to unsafe neighborhoods and a lack of green space,^33^ although most studies evaluating effects of built environment on physical activity originate from North America.^34^ The generalizability of these data to a UK context has been questioned, and further research is needed to understand these processes in the UK.^35^

The fact that a consistent association of family affluence with these health behaviors is observed, despite substantial variability in underlying causes, is consistent with a view of SES as a “fundamental cause” of health.^20^ That is, as risk factors for ill health become clear, patterning by SES follows, even where those risk factors have different causes. The finding of higher alcohol consumption among adolescents from more affluent families runs counter to the dominant trend for more healthy behaviors in these groups, and has been reported previously in 1 UK study, which argues that increased family affluence may increase the availability of alcohol in the family.^36^ If explained by home alcohol availability, it may be that children from more affluent families drink more regularly, but in moderate amounts, under parental supervision.

In most studies included in Hanson and Chen's aforementioned review, SES was evaluated only at the family level. Many studies which incorporated measures of SES at the school level typically either did not simultaneously measure family-level SES^37^ or conducted only single-level regression analyses.^38^ This study is novel in that it contributes evidence that, when using multilevel analysis methods which simultaneously examine variance at school and individual levels, school-level affluence is independently associated with health behaviors after adjusting for family affluence. All behaviors except physical activity were more healthful in more affluent schools.

School socioeconomic environments may affect health behavior via a range of mechanisms. Social networks and peer influences have been shown to be significantly related to adolescents' smoking^39^ and dietary behaviors,^32^ with peer norms gradually replacing parental norms as key influences upon behavior throughout adolescence.^32^ Children who attend more affluent schools might be exposed to fewer peers who smoke, or more peers who consume healthier diets. Furthermore, affluent parents who possess more flexible resources^20^ may apply their greater social influence to affect delivery of school services which impact health.

Findings were also consistent with the hypothesis that school and family affluence interact to shape health behaviors. Whereas behaviors were healthier in more affluent schools, a significantly stronger association of family SES with health behavior was observed within these schools. For example, attending a more affluent school was associated with reduced risk of smoking only among children from affluent families. Although attending a more affluent school was associated with higher fruit and vegetable consumption for children of all levels of family affluence, between-school differences were substantially smaller among children from poorer families. In relation to physical activity, the smaller overall between-school variance by comparison to other behaviors perhaps reflects in part the standardization of physical education curriculum throughout the UK. However, findings suggest that attending a more affluent school might be harmful for children from poorer families, with children from poorer families less likely to be physically active if they attend a more affluent school.

These interaction effects may reflect conflicts between norms developed in the home environment and those observed in school among children from poorer families attending schools with more affluent intakes. As described above, while attending a school where overall peer norms are healthier may be expected to improve health behaviors, poorer children may find the norms and values of such schools less congruent with those they encounter outside of school.^22^ Hence, they may be more likely to disengage with mainstream norms and cultures of such schools, and to associate with subgroups of children from similar backgrounds. By contrast, children from more affluent families, but who attend more deprived schools, may be adversely influenced by greater exposure to mainstream norms for less healthy behaviors.

The finding that the least active children were from poorer families, but attending affluent schools, also may reflect disengagement from school culture. However, Green^40^ argues that parents exert a strong influence on the types of physical activity opportunities delivered within schools; schools with a more affluent intake may experience greater parental pressures to offer extracurricular activities in which children from poorer families cannot afford to participate. Hence, more affluent parents perhaps apply flexible resources to influence the delivery of school services in ways which disadvantage children from less affluent families.^20^ It is also possible that children from poorer families may have to travel further to attend such schools, limiting time for physical activity outside of school.

### Limitations

Key strengths of this study include use of a large, nationally representative sample, meaning that results can be generalized reliably, although further analysis of generalizability beyond Wales would be a useful direction for future research. Limitations include that all measures of health behavior were based on self-reports. Objective measures, such as accelerometers, would be too resource intensive, and while increasing precision, may lower response rates and introduce response biases. The cross-sectional design means that cause and effect cannot be established. It is possible that associations of school-level affluence with health behaviors were in part confounded by area-level affluence. Whereas models adjusted for children's reports of the perceived affluence of their area, no validated measure of area-level deprivation was available. Potential measures include the Welsh Index of Multiple Deprivation, which if linked to individual child responses, could facilitate robust analysis of associations of SES with health behaviors at the school, family, and neighborhood levels simultaneously. The study also assesses family affluence on the basis of material consumption. This may lead to misclassification of some families that are affluent, but that save rather than spend. However, the strong correlation between FSM and family affluence reported in this study suggests that FAS has good validity in this sample. Only one dimension of family-level SES was examined, whereas other studies have included measures such as parents' education or occupation.^41^

### Conclusions

School and family affluence are independently, and in combination, associated with health behaviors of schoolchildren in Wales. Overall, children who attend more affluent schools are less likely to smoke and more likely to eat fruits and vegetables. However, within school, inequalities with respect to these behaviors are greater in more affluent schools. That is, family affluence is more strongly associated with health behavior in more affluent schools than in poorer schools. Whereas no overall association of school affluence with physical activity was observed, SES gradients within more affluent schools are again substantially greater, with children from poorer families least likely to be active if attending a more affluent school.

## Implications for School Health

Findings have a number of implications for policy, practice, and future research. At present, little is known regarding the effects of school-based interventions on socioeconomic inequalities. In 2 Cochrane reviews of school-based interventions, socioeconomic inequalities were neither mentioned^11^ nor examined in any primary studies included.^42^ To build this evidence base, evaluations of school-based interventions should examine whether effects are patterned by school and family-level SES. Qualitative inquiry also could help in developing an understanding of the mechanisms through which school and family-level SES interact, and the different ways in which SES groups perceive and interact with schools and school-based interventions. As educational outcomes often are seen as the main priority for schools, it can be difficult to engage schools in action to improve health.^43^ However, there is a growing body of evidence linking improved health behaviors with better educational outcomes,^43^–^46^ whereas inequalities have been observed consistently in educational outcomes.^47^ Hence, there is a need for greater engagement between researchers and school staff to support schools in developing, implementing, and evaluating action plans that may reduce inequality in health and education outcomes.^48^

Findings also have important implications for the nature of targeting of school-based health interventions. Whereas Hanson and Chen suggest that interventions should target more deprived children, their review does not distinguish between family- and school-level SES.^8^ School-level SES should not be seen as a simple aggregation of individual-level SES, but as a characteristic of the setting. Strategies to reduce health inequalities need to target socioeconomic factors at multiple levels of the socioecological framework.^18^,^19^ Interacting effects of school- and family-level factors suggest that targeting intervention toward more deprived schools may fail to address substantial inequalities within more affluent schools. Targeting more deprived families may fail to improve behaviors of children from affluent families, attending more deprived schools. Universal interventions that have greater effects among children from poorer backgrounds^14^ may be a more effective means of reducing inequalities.^2^

### Human Subjects Approval Statement

The HBSC survey received approval from the Cardiff University School of Social Sciences Research Ethics Committee.
